# The structure of a family 168 glycoside hydrolase from the marine bacterium *Muricauda eckloniae*

**DOI:** 10.1107/S2053230X2500425X

**Published:** 2025-05-27

**Authors:** Emily Knudson-Goerner, Alisdair B. Boraston

**Affiliations:** a Biochemistry and Microbiology University of Victoria PO Box 1700 STN CSC Victoria BCV8W 2Y2 Canada; University of York, United Kingdom

**Keywords:** glycoside hydrolases, fucoidanases, marine bacteria, *Muricauda eckloniae*

## Abstract

The genome of the marine bacterium *Muricauda eckloniae* sp. DK169 contains an extensive polysaccharide-utilization locus that targets fucoidan from brown algae. Within this locus is a gene that encodes a putative fucoidan-degrading glycoside hydrolase (locus tag AAY42_01205) assigned to glycoside hydrolase family 168, which we call *Me*GH168. We present the 2.0 Å resolution X-ray crystal structure of *Me*GH168, demonstrating a (β/α)_8_-barrel fold. The eight loop regions joining each α-helix and β-strand surround the catalytic groove. A comparison with the structure of a GH168, Fun168A, in complex with a fragment of fucoidan (PDB entry 8ya7) revealed conservation of key residues in the catalytic site. However, structural variation in positive-subsite loop regions may recontour the active site to create differences in substrate specificity between the two GH168s. The present data provide additional structural insights into the GH168 family, particularly expanding on sequence and structure conservation (and the lack thereof) in relation to substrate interactions.

## Abstract 2

The structure of a putative fucoidan-degrading glycoside hydrolase assigned to glycoside hydrolase family 168 reveals a (β/α)_8_ fold. The catalytic machinery and potential substrate specificity were investigated through a structural comparison with a Fun168A oligosaccharide complex.

## 1. Introduction

Species in the *Muricauda* genus are distributed in diverse marine environments ranging from coastal ecosystems to deep-sea hydrothermal fields (Cao *et al.*, 2023 ▸). *M. eckloniae* sp. DK169 was isolated from the rhizosphere of *Ecklonia kurome*, a marine alga found on the coast of Dokdo Island, Korea (Bae *et al.*, 2007 ▸). Accordingly, *M. eckloniae* primarily colonizes macroalgal surfaces and has garnered increasing interest due to the presence of genomic regions which encode unique carbohydrate-active enzymes (CAZymes) capable of degrading algal polysaccharides (Tran *et al.*, 2022 ▸; Mikkelsen *et al.*, 2023 ▸). Algal polysaccharides often possess chemically diverse and sophisticated structures; therefore, algal-specific CAZymes often have structural characteristics that impart high substrate specificity to effectively process polysaccharides (Bäumgen *et al.*, 2021 ▸; Arnosti *et al.*, 2021 ▸; Sarkar *et al.*, 2024 ▸). As a result, chemically complex algal polysaccharide degradation has yet to be thoroughly characterized, particularly that of sulfated fucans such as fucoidan.

Fucoidan is a complex polysaccharide that is found in several brown algae species. It is primarily composed of an α-1,3-linked l-fucose backbone or an alternating α-1,3/1,4 backbone, depending on the source species (Mikkelsen *et al.*, 2023 ▸; Ale *et al.*, 2011 ▸; Chen *et al.*, 2024 ▸; Buck-Wiese *et al.*, 2023 ▸; Kusaykin *et al.*, 2015 ▸). Additionally, heavy sulfation is present primarily on C2 and/or C4 for most or all fucose residues. Along with varying backbone linkage and sulfation patterns, the complexity of fucoidans is further established by species-dependent branching patterns, occasional acetylation and the presence of varying sulfated monosaccharides in its backbone. As a result, different fucoidan-targeting enzymes, or fucoidanases, must accommodate a wide variety of substrate configurations to process the complex structure of fucoidan. However, relatively few fucoidanases have been characterized to date, and thus further investigation of their unique abilities to recognize a structurally heterogenous substrate is required.

The fucoidan-degradation locus of *M. eckloniae* sp. DK169 encodes 25 proteins associated with fucoidan depolymerization, transport and assimilation (Tran *et al.*, 2022 ▸; Mikkelsen *et al.*, 2023 ▸). This fucoidan-utilizing locus is mostly comprised of glycoside hydrolases and sulfatases, revealing a wide variety of substrate specificities required for depolymerization. Amongst these is a gene encoding a member of glycoside hydrolase family 168, which we call *Me*GH168, which is of particular interest due to its potential in initiating fucoidan degradation. In general, the inferred catalytic mechanism of GH168s is a two-step retaining mechanism utilizing two carboxylic acid-containing amino acids as nucleophile and acid–base residues (Shen *et al.*, 2020 ▸; Silchenko *et al.*, 2023 ▸; Chen *et al.*, 2024 ▸). Recently, GH168 enzymes have garnered particular interest, with an increasing number of structures characterized. Of these, the structure of FunGH168 from *Wenyingzhuangia fucanilytica*, which is trapped in complex with a fucoidan-hydrolysis product, has demonstrated the molecular basis of its endo-hydrolytic activity on α-1,3-l-fucose linkages (Shen *et al.*, 2020 ▸; Chen *et al.*, 2024 ▸). Here, we report the structure of *Me*GH168 and use a comparison of the structure with the FunGH168 product complex to reveal the conservation of catalytic residues and some subsite-binding motifs. However, major structural differences suggest potential differences in substrate specificity.

## 2. Materials and methods

### 2.1. Macromolecule production

The gene encoding *Me*GH168 from *M. eckloniae* sp. DK169 (locus tag AAY42_01205; GenBank KQC28672.1) was obtained as a synthetic gene comprising an in-frame fusion with an N-terminal His_6_-tag to generate pET-28a-MeGH168 (Hobbs *et al.*, 2019 ▸). The predicted secretion signal peptide was omitted, resulting in the amino-acid boundaries 19–375. Protein production and purification were performed as described previously, utilizing Ni^2+^–NTA immobilized metal-affinity chromatography followed by size-exclusion chromatography using a Sephacryl S-200 column (GE Healthcare) pre-equilibrated with 20 m*M* Tris–HCl pH 8.0 containing 500 m*M* sodium chloride (Hobbs *et al.*, 2019 ▸). The purified protein was concentrated to ∼10–11 mg ml^−1^ using a stirred-cell ultrafiltration device (Amicon) with a 10 000 Da molecular-weight cutoff membrane (Millipore) for crystallization. Macromolecule-production information is summarized in Table 1 ▸.

### 2.2. Crystallization

Crystallization screening was conducted with the commercially available screens Index, PEG/Ion and Crystal Screen (Hampton Research) by sitting-drop vapour diffusion at 291 K with a 1:1 ratio of protein solution (11 mg ml^−1^ in 20 m*M* Tris–HCl pH 8.0, 500 m*M* sodium chloride) and crystallization solution (0.5 µl each). A crystal grown in 20%(*w*/*v*) PEG 3350 with 8%(v/*v*) Tacsimate pH 7.0 was harvested from the screen and used to collect diffraction data. Crystallization information is summarized in Table 2 ▸.

### 2.3. Data collection and processing

The crystal was cryoprotected in crystallization solution with 20%(*v*/*v*) ethylene glycol and immediately cooled in liquid nitrogen for storage and shipping. Diffraction data were collected on the CMCF-ID beamline at the Canadian Light Source (CLS). Data were integrated, scaled and merged with *XDS* and *AIMLESS* (Kabsch, 2010 ▸; Evans & Murshudov, 2013 ▸; Agirre *et al.*, 2023 ▸).

Data-collection and processing statistics are summarized in Table 3 ▸.

### 2.4. Structure solution and refinement

The crystal structure of *Me*GH168 was solved by molecular replacement using *Phaser* (McCoy *et al.*, 2007 ▸) and an *AlphaFold*-generated model of *Me*GH168 (Jumper *et al.*, 2021 ▸; Varadi *et al.*, 2022 ▸). *Coot* was used to manually correct the model and *phenix.refine* was used for refinement of the experimental model and the addition of water molecules (Liebschner *et al.*, 2019 ▸; Emsley & Cowtan, 2004 ▸). Refinement procedures were monitored by flagging 5% of all observations as ‘free’ (Brünger, 1992 ▸). Model validation was performed with *MolProbity* (Chen *et al.*, 2010 ▸). A summary of the model statistics is shown in Table 4 ▸.

## 3. Results and discussion

### 3.1. Overall structure of *Me*GH168

*Me*GH168 crystallized in space group *P*22_1_2_1_ with unit-cell parameters *a* = 47.27, *b* = 120.93, *c* = 163.85 Å. Analysis using *MATTHEWS_COEF* (Kantardjieff & Rupp, 2003 ▸) showed two protein molecules in the asymmetric unit with a Matthews coefficient of 2.65 Å^3^ Da^−1^ and a solvent content of 53.67%. *PISA* analysis indicated that dimers of the protein are unlikely to form in solution (Krissinel & Henrick, 2007 ▸). Recombinant protein residues 20–375 were modelled and refined; no gaps were present in the backbone.

**Figure 1:**
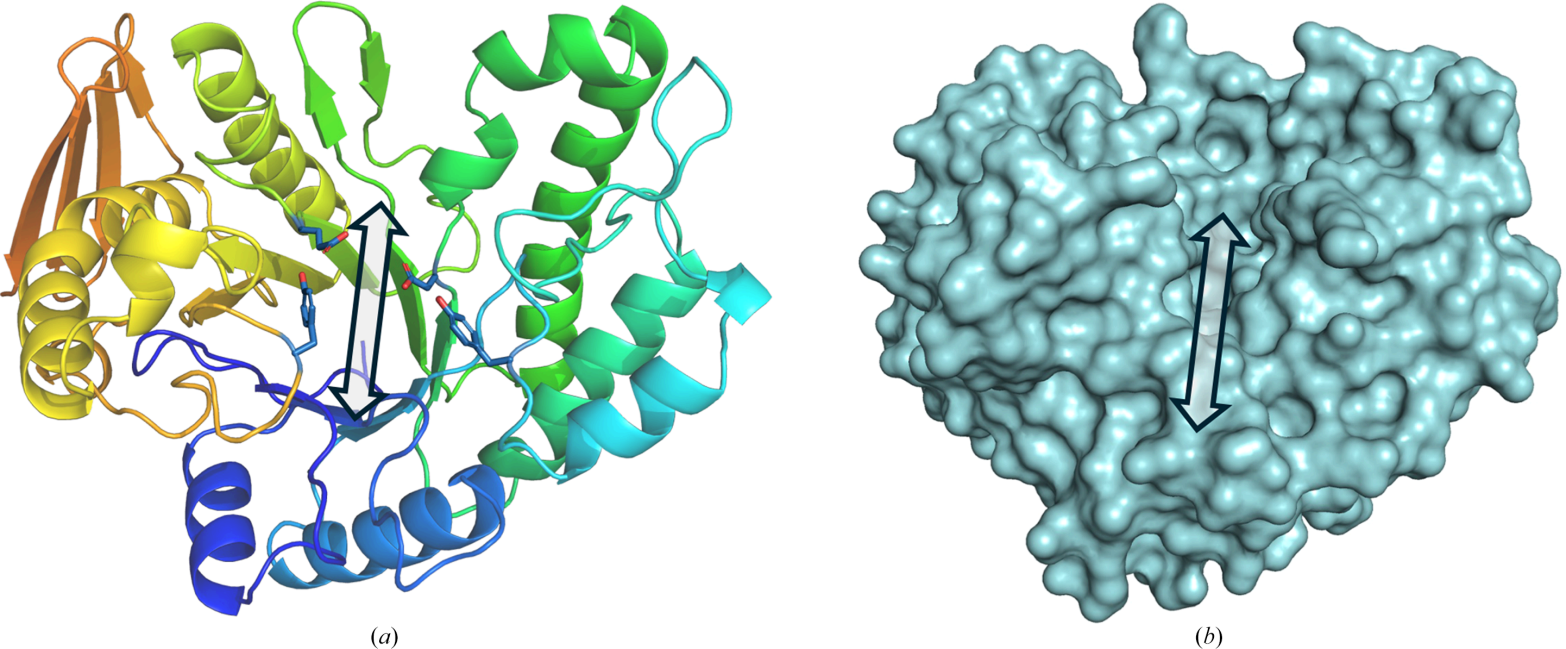
Overall structure of *Me*GH168. (*a*) Cartoon representation of *Me*GH168. Each β–α region is shown in different colours, with the (β/α)_8_ barrel sequentially shown in purple to orange and the C-terminal β-sheet fold in red. Aromatic and predicted catalytic residues in the active-site cleft are shown in blue stick representation. (*b*) Surface representation of *Me*GH168. Arrows in both panels indicate the catalytic groove.

The structure of *Me*GH168 is composed of a (β/α)_8_ triosephosphate isomerase (TIM) barrel fold, with eight α-helices and eight parallel β-strands alternating at the core of the enzyme (Fig. 1 ▸*a*). Connecting each α-helix and β-strand are eight unique loop regions. Additionally, an antiparallel β-sheet fold, consisting of four β-strands, is present in the C-terminal region. Based on an estimation of the active-site length (approximately 25 Å), determined by cleft topology (Fig. 1 ▸*b*), in combination with the distribution of aromatic residues (Fig. 1 ▸*a*), we expect approximately five subsites to be present, with either two or three subsites on either side of the scissile bond.

**Figure 2:**
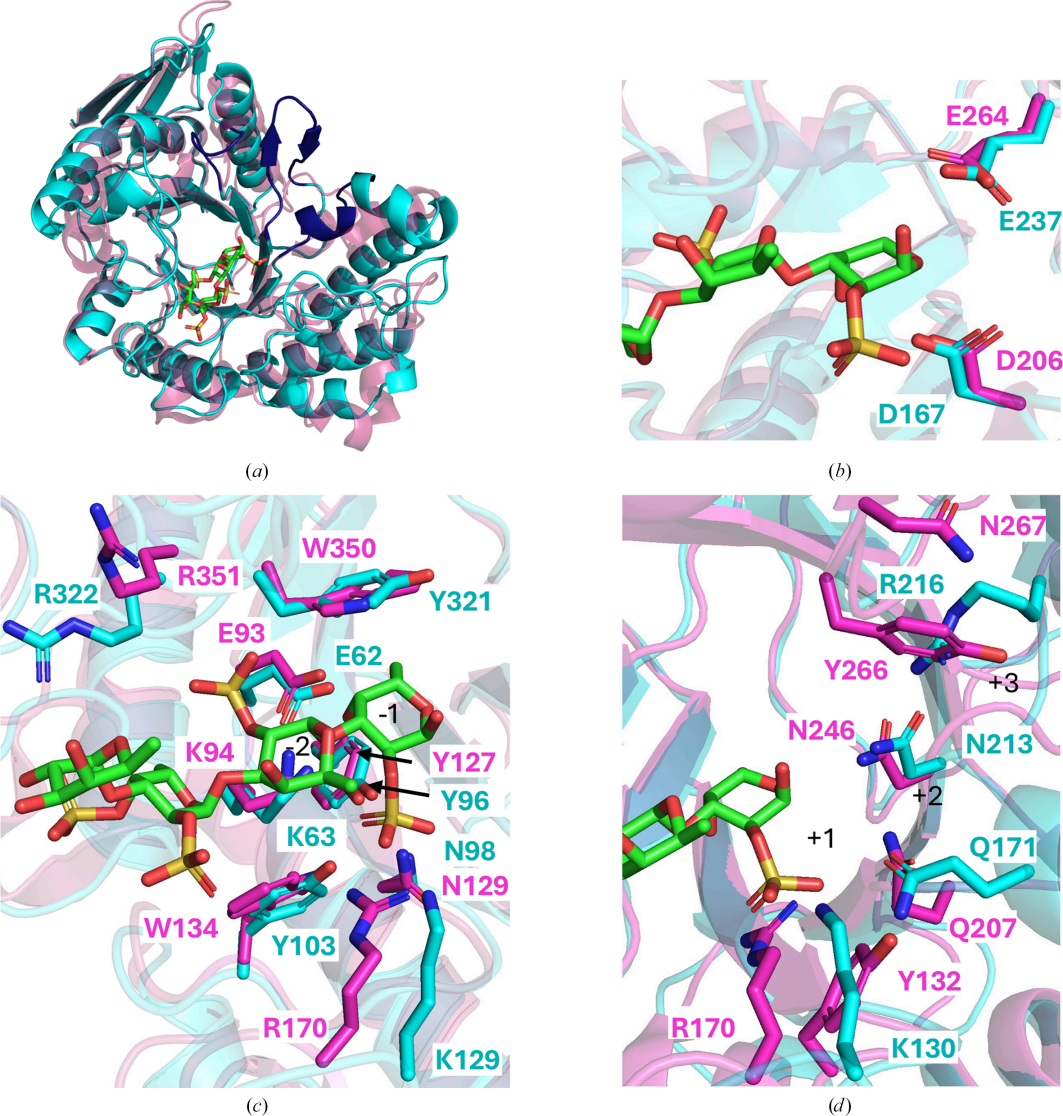
Structural alignment of *Me*GH168 with the Fun168A product complex provides insight into its active site. (*a*) Complete alignment of *Me*GH168 (cyan) and Fun168A (PDB entry 8ya7, pink) shown in cartoon representation. *Me*GH168 loop regions that are structurally variable in comparison to Fun168A are shown in navy. (*b*) Active-site catalytic residues of *Me*GH168 (cyan) compared with Fun168A (PDB entry 8ya7, pink). (*c*) Residues in negative subsites (−1, −2) of *Me*GH168 (cyan) compared with Fun168A (PDB entry 8ya7, pink). (*d*) Residues in suggested positive subsites (+1, +2, +3) of *Me*GH168 (cyan) compared with Fun168A (PDB entry 8ya7, pink). The fucoidan tetrasaccharide ligand is represented in green sticks.

Structural similarity results from a *DALI* server search yielded Fun168A from *W. fucanilytica* as the top hit [PDB entry 8ya7; *Z*-score and root-mean-square deviation (r.m.s.d.) of 40.5 and 2.3 Å, respectively; 29% amino-acid sequence identity]. The general structure of Fun168A has the same (β/α)_8_ TIM-barrel fold. The structures of *Me*GH168 and Fun168A both have eight loop regions connecting the barrelling α-helices and parallel β-strands. Compared with Fun168A (PDB entry 8ya7), *Me*GH168 shows some structural conservation in loop regions near the front of the catalytic groove (for reference: the loop regions to the left of the Fun168A fucoidan ligand in Fig. 2 ▸*a*). However, the loop regions at the back of the catalytic groove have high variability, suggesting some possible contributions to differences in substrate recognition. In particular, an antiparallel β-hairpin in the predicted positive-subsite region of *Me*GH168 is likely to have a profound effect on reshaping the active site of this enzyme (Fig. 2 ▸*a*). It may promote the recognition of a different fucoidan conformation resulting from an alternate backbone structure, such as the presence of α-1,4-linkages. Notably, the β-hairpin could convert this particular enzyme to an exo-activity, rather than the endo-activity observed for Fun168A, by potentially preventing the accommodation of larger substrates.

### 3.2. Structure of the catalytic groove

The structure of Fun168A was determined as a product complex bound to α-l-Fuc*p*-1,3-α-l-Fuc*p*2,4(OSO_3_^−^)-1,3-α-l-Fuc*p*2(OSO_3_^−^)-1,3-α-l-Fuc*p*2(OSO_3_^−^) with ligand inter­actions through the −1 to −2 subsites (Chen *et al.*, 2024 ▸). The −3 and −4 subsites displayed no ligand interactions as these fucosyl residues did not make direct interactions with the enzyme, making it unclear whether there truly are −3 and −4 subsites; therefore, we opted to omit analysis of these subsites. A comparison of *Me*GH168 with this structure revealed conservation of the catalytic residues (Asp167 and Glu237 in *Me*GH168) in structurally conserved loop regions in the central base of the catalytic groove (Fig. 2 ▸*b*). Based on the retaining mechanism of GH168 enzymes, Asp167 is predicted to function as the nucleophile and Glu237 as the acid/base.

Ligand binding in Fun168A involves salt bridges and hydrogen-bond interactions with sulfate groups specifically, allotting importance to the residues involved in these interactions (Chen *et al.*, 2024 ▸). As demonstrated in Fig. 2 ▸(*c*), the Fun168A −1 and −2 subsite residues are generally well conserved within *Me*GH168. Nonconserved residues in *Me*GH168 still possessed chemically similar side chains, which could facilitate the same CH–π stacking and salt-bridge interactions as observed in Fun168A.

Putative +1, +2 and +3 subsites in Fun168A were identified by molecular-docking studies (Chen *et al.*, 2024 ▸). It should be noted that the authors experimentally confirmed +1 site specificity in Fun168A; however, the +2 and +3 subsite interactions remain putative. The potential positive subsite residues in *Me*GH168 were identified based on their spatial proximity to corresponding subsites in Fun168A. In Fun168A, +1 subsite specificity was attributed to the residues Arg170, Tyr266 and Gln207, which appears to impart strict binding for nonsulfated fucose (Chen *et al.*, 2024 ▸). The *Me*GH168 residues in the closest proximity are Lys130, Arg216 and Gln171, respectively (Fig. 2 ▸*d*). This suggests that the interactions of these residues would likely be similar; however, the presence of Arg216 in comparison to Tyr266 in Fun168A may reflect a somewhat altered set of interactions in this subsite. Residues in the +2 subsite (Arg170, Tyr132 and Asn267 in Fun168A) were not conserved in *Me*GH168, with the exception of Asn213. Similarly, Asn267, His209 and Tyr266, present in the suggested +3 subsite, were not present in *Me*GH168. The lack of structural and sequence conservation in the anticipated positive subsites of *Me*GH168, combined with presence of a large β-hairpin loop insertion (Fig. 2 ▸*a*), suggests that this enzyme may be able to accommodate substrates with alternative chemical characteristics in comparison to Fun168A. However, further investigations into the activity of this enzyme are required to determine the exact substrate specificity.

## 4. Conclusion

The structure of *Me*GH168 from the marine bacterium *M. eckloniae* sp. DK169 belongs to the sparsely characterized GH168 family. The conservation of catalytic and negative-subsite residues suggests similarities to the retaining hydrolysis mechanism of similar fucoidan substrates observed in the GH168 family. However, structural variation in positive subsites located in loop regions demonstrating high structural variability suggests potential differences in substrate specificity in *Me*GH168 compared with Fun168A.

## Supplementary Material

PDB reference: *Me*GH168, 9nhf

## Figures and Tables

**Table 1:** Macromolecule-production information

Source organism	*Muricauda eckloniae* DK169 (strain DOKDO 007T)
DNA source	Synthetic DNA construct
Expression vector	pET-28a
Expression host	*Escherichia coli*
Complete amino-acid sequence of the construct produced†	**MGSSHHHHHHSSGLVPRGSHM**QQEYYPNFSWDKVPVAFHFGKRDGLMTKDEAKFVTSRSNFIVLEKAHGAPDYEYTEDAIAKEARKLKKLNPGMKVIFYWNSFLDYNMYKAHEVYQNHPQWWLRKQDGELDFKNKGLKRYDLSNPKVRDWWTDVAKNEIVNGSTDGIFMDAFIQVSNPANIKLWGQKKYNDIQQGLKDLIKETREKLGDDKLIVYNGIRSTFQRNVGNNFPDYTDVVMIEHFGHFASTSKESMLTDIQEMEKAGKSGKIVVFKAWPGFAWIDKEAMSKPYVEKQKIAKNSITFPLAAFLAGAQEHSYFIYNWGYRMEMGCLEWYPEFDKPLGKPLNDMVINGWVLTREYEHALVWVNLETNEAKINWK

† The His_6_-tag sequence is shown in bold.

**Table 2:** Crystallization information

Method	Vapour diffusion, sitting drop
Temperature (K)	291
Protein concentration (mg ml^−1^)	11
Buffer composition of protein solution	20 m*M* Tris–HCl pH 8.0, 500 m*M* sodium chloride
Composition of reservoir solution	20%(*w*/*v*) PEG 3350, 8%(*v*/*v*) Tacsimate pH 7.0
Volume and ratio of drop	1 µl, 1:1
Volume of reservoir (µl)	50

**Table 3:** Data collection and processing Values in parentheses are for the outer shell.

Diffraction source	CLS
Wavelength (Å)	0.954
Temperature (K)	100
Detector	Dectris EIGER X 9M
Rotation range per image (°)	0.2
Total rotation range (°)	360
Exposure time per image (s)	0.5
Space group	*P*22_1_2_1_
*a*, *b*, *c* (Å)	47.27, 120.93, 163.85
α, β, γ (°)	90, 90, 90
Mosaicity (°)	0.1
Resolution range (Å)	97.31–2.00 (2.05–2.00)
Total No. of reflections	493807
No. of unique reflections	64544
Completeness (%)	100.0 (100.0)
Multiplicity	7.6 (8.0)
〈*I*/σ(*I*)〉	12.8 (2.4)
*R*_p.i.m._	0.051 (0.364)
*R*_meas_	0.142 (1.045)
CC_1/2_	0.997 (0.764)
Overall *B* factor from Wilson plot (Å^2^)	23.59

**Table 4:** Structure solution and refinement Values in parentheses are for the outer shell.

Resolution range (Å)	48.650–2.000 (2.071–2.000)
Completeness (%)	99.97
No. of reflections, working set	64518 (6356)
No. of reflections, test set	3186
Final *R*_work_	0.1989 (0.2001)
Final *R*_free_	0.2327 (0.2966)
No. of non-H atoms	
Protein	2952
Ligand	1 [EDO]
Water	842
Total	6767
R.m.s. deviations	
Bond lengths (Å)	0.002
Angles (°)	0.49
Average *B* factors (Å^2^)	
Protein	25.93
Ligand	31.71 [EDO]
Water	35.82
Ramachandran plot	
Favoured regions (%)	98.45
Allowed (%)	1.55
Outliers (%)	0.00
PDB code	9nhf
